# Letter to the editor regarding the article ‘Coronary spasm as a manifestation of allergic reaction to contrast material: a case report of Kounis syndrome’ by Zmolik *et al.*

**DOI:** 10.1093/ehjcr/ytaf578

**Published:** 2025-11-05

**Authors:** Francisco Vega

**Affiliations:** Department of Allergy, Hospital Universitario de la Princesa, Instituto de Investigación Sanitaria (IIS-Princesa), Diego de Leon 61, 28006 Madrid, Spain

I read with great interest the article by Zmolik *et al*., ‘Coronary spasm as a manifestation of allergic reaction to contrast material: a case report of Kounis syndrome’,^1^ which describes a rare hypersensitivity reaction to iopromide in a patient undergoing CT imaging, published in your esteemed journal.

An interesting aspect of this case report is the potential role of concomitant pembrolizumab administration in triggering hypersensitivity reaction (HS). Pembrolizumab is an immunotherapeutic agent from the group of immune checkpoint inhibitors, composed of monoclonal antibodies that block surface proteins involved in maintaining immune homeostasis. Some of the best-known immune checkpoint proteins include PD-1, PD-L1, CTLA-4, and LAG-3. Pembrolizumab specifically targets PD-1, a surface protein expressed on T cells that binds to its natural ligand PD-L1, commonly found on tissue cells.

The physiological role of this immune checkpoint is to prevent autoimmunity: when a T lymphocyte recognizes these surface proteins, it identifies the cell as self and does not initiate an immune attack. Certain neoplastic cell lines exploit this mechanism by overexpressing these proteins, thereby evading immune surveillance, and then, T cells fail to recognize them as abnormal and do not destroy them. Checkpoint inhibitors block this protective mechanism, restoring the immune system’s ability to recognize and eliminate tumour cells. However, this immune dysregulation may also facilitate sensitization to other concurrently administered medications, as previously described.^2^ This immunomodulatory action might have contributed to the development of a HS to contrast media (CM) in this patient who had previously tolerated.

During the emergency coronary angiography performed to confirm the diagnosis of Kounis syndrome (KS), a localized coronary vasospasm was observed. Although this was initially attributed to mechanical stimulation from catheter contact, it is also possible that a new HS to the CM used in this procedure (iodixanol) played a role, given the known high and variable cross-reactivity among CM. In this context, it is important to note that the route of CM administration can influence both the type and distribution of hypersensitivity symptoms. Intravenous administration tends to result in generalized symptoms, whereas intra-arterial administration is more likely to produce localized manifestations within the perfused vascular territory. Van der Molen *et al*.^3^ have reported 41 cases of KS induced by CM, over 25% of which were triggered by intra-arterial administration. Notably, in 37% of these cases—similar to the one reported by Zmolik *et al*.—a second KS episode occurred during the emergency coronary angiography following an initial episode triggered by intravenous CM.

Finally, the authors highlight the challenges of conducting contrast-enhanced oncological follow-up in this patient due to the HS to CM. In this regard, I would like to point out that in recent years, significant progress has been made in identifying safe alternative CM for such cases. This approach is also recommended by the European Academy of Allergy and Clinical Immunology (EAACI).^4^ Selection of an alternative CM can be guided by classification of cross-reactivity among them^5^ or, more reliably, through a comprehensive allergy work-up that includes skin testing and controlled provocation tests.^6^

**Funding:** None declared.

## Data Availability

There are no new data associated with this article.
